# Editorial: Regulation of horticultural fruits and vegetables quality: internal or external factors

**DOI:** 10.3389/fpls.2023.1264533

**Published:** 2023-08-21

**Authors:** Junbei Ni, Minjie Qian, Jianzhao Li, Ramón Gerardo Guevara-González

**Affiliations:** ^1^ Zhejiang University, Hangzhou, China; ^2^ Hainan University, Haikou, China; ^3^ Ludong University, Yantai, China; ^4^ Center for the Applied Research in Biosystems (CARB-CIAB), School of Engineering, Autonomous University of Querétaro, El Marques, Querétaro, Mexico

**Keywords:** fruit quality, stress management, omic studies, vegetables production, plant biotechnology strategy

Current horticultural production requires high-quality standards to improve the functionality of fruits and vegetables. For this reason, several research strategies have originated at pre- and postharvest production levels. The present Research Topic aimed to increase the level of understanding about the influence of internal and external factors on the quality of fruits and vegetables. This Research Topic shows five interesting investigations related to generating knowledge that might be useful for fruits and vegetable production with high-quality standards, as abovementioned. From the application of light at the postharvest stage in mango to improve the metabolite profile (Zhu et al.) to the discovery of molecular aspects related to the role of long non-coding RNAs during fruit development in highbush blueberry (Li et al.), this research displayed clear insights that adequate management of strategies using external environmental stimuli in horticultural production should effectively impact internal features that might increase the quality of fruits and vegetables.

Thus, the novel knowledge related to changes in gene expression associated with sugar contents in litchi fruit (Peng et al.) and strawberry (Wu et al.), as well as the association of specific QTLS with sugar content during *P. pyrifolia* fruit ripening (Jiang et al.), are clear examples that these strategies are necessary to reach the quality standards of fruit and vegetables customers are requesting nowadays.

In summary, it is clear that unraveling molecular aspects related to a phenotype of interest in plant science is highly important to propose future rationale strategies to improve quality in horticultural production. Noteworthy mentioning that future research on producing high-quality fruits and vegetables (including their nutraceutical and functional properties) will be an imperative requirement of horticultural production based on the current needs of customers. Additionally, sustainable strategies for managing external stimuli to reach this goal *via* the biostimulation/elicitation of interesting phenotypes in horticultural products will also be necessary in the current climate change scenario worldwide.

## Author contributions

RG-G: Writing – original draft. JN: Writing – review & editing. MQ: Writing – review & editing. JL: Writing – review & editing.

